# Glycerol-mediated improvement of heterologous aurachin D production in *E. coli*

**DOI:** 10.1007/s00253-026-13890-2

**Published:** 2026-05-30

**Authors:** Jonas Korb, Büsra Demir, Kai Graw, Markus Nett

**Affiliations:** Department of Biochemical and Chemical Engineering, TU Dortmund University, Dortmund, Germany

**Keywords:** Aurachin, Biotransformation, *Escherichia coli*, Glycerol, Whole-cell biocatalysis

## Abstract

**Abstract:**

Neglected tropical diseases and the increasing prevalence of antibiotic resistance in nosocomial infections represent a major challenge in medicine. Intensive research into bioactive compounds has identified the myxobacterial natural product aurachin D as promising drug candidate due to its potent antibiotic and antiprotozoal properties. The biosynthesis of aurachin D from 2-methyl-1*H*-quinolin-4-one (MQO) is catalyzed by the membrane-bound farnesyltransferase AuaA. Recently, this enzymatic conversion was reconstructed in the heterologous host *Escherichia coli*, yielding an aurachin D titer of 17 mg L^−1^. In this study, we investigated the influence of the medium on aurachin D biosynthesis in *E. coli* in order to improve the product titer. Our analyses revealed that the glycerol concentration has a major impact on both the growth of the production host and the biocatalytic formation of aurachin D. After switching to a glycerol-based minimal medium and adjustment of the MQO precursor concentration, an aurachin D titer of 124 mg L^−1^ was achieved in a microbioreactor system, highlighting the superior performance of minimal over complex medium as well as the beneficial effect of glycerol compared to glucose. Further studies were conducted to elucidate the mechanism underlying glycerol’s effects. While improved growth and substrate solubilization were found to be insignificant for increasing the product titer, it was demonstrated that glycerol supplementation positively influenced the expression of the enzyme AuaA. Using a folding reporter, we were able to show that the availability of correctly folded AuaA increased until growth defects of the host, presumably resulting from osmotic stress, began to appear.

**Key points:**

• *Product toxicity is negligible in the production of aurachin D using E. coli*

• *The media supplement glycerol improves the availability of the enzyme AuaA*

• *Aurachin D titers increase after switching to a minimal medium*

**Graphical abstract:**

**Figure:**
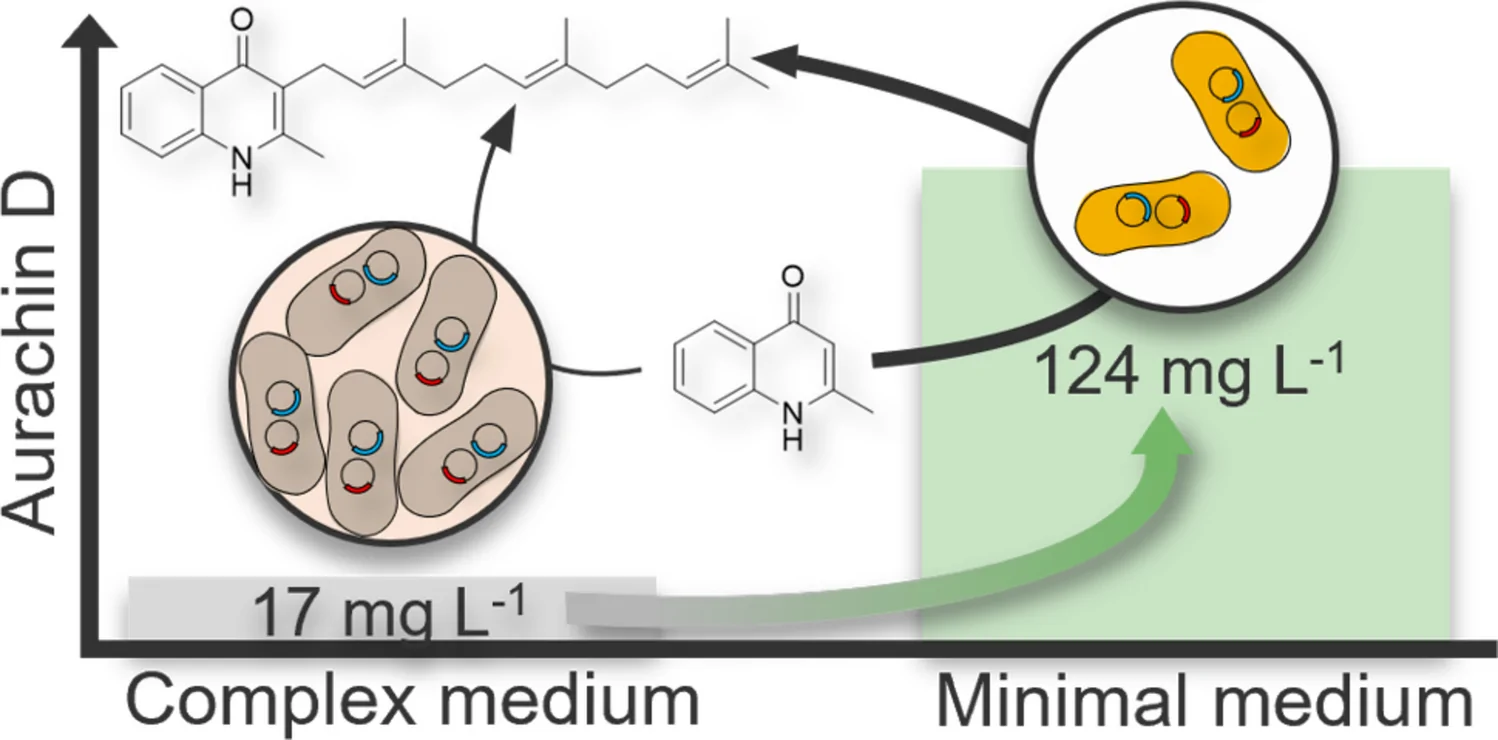

**Supplementary Information:**

The online version contains supplementary material available at 10.1007/s00253-026-13890-2.

## Introduction

The aurachins constitute a family of farnesylated quinolone alkaloids that were originally discovered in myxobacteria of the genus *Stigmatella* (Kunze et al. 1987). Due to their structural relatedness to menaquinones and ubiquinones, the aurachins interfere with electron transport processes in the respiratory chain (Kruth and Nett 2023). Of particular interest in this context is aurachin D, which was identified as a potent inhibitor of the terminal oxidase cytochrome *bd* (Grauel et al. 2021). Since the *bd* oxidase occurs exclusively in the electron transport chains of bacteria and archaea, aurachin D represents a promising lead structure for drug development. Various attempts were made to further increase the pharmacological selectivity of aurachin D by targeted modifications (Radloff et al. 2021; Lawer et al. 2022). Recently, aurachin D was also reported to possess considerable activity against *Leishmania donovani*, a human blood parasite causing the severe and potentially fatal disease visceral leishmaniasis (Kruth et al. 2023).

**Fig. 1:**
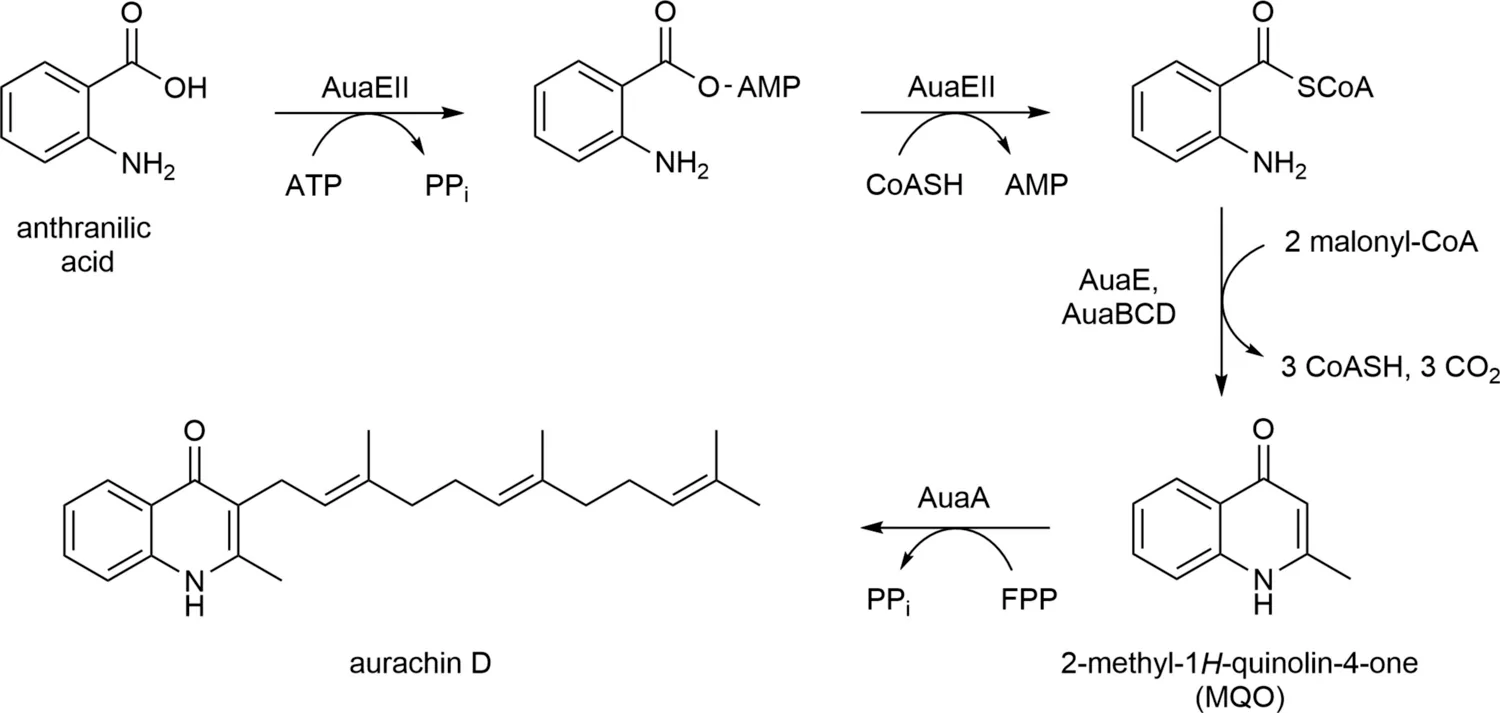
Biosynthetic pathway to aurachin D in *Stigmatella aurantiaca*. Abbreviations: CoASH, coenzyme A; FPP, farnesyl pyrophosphate; PP_i_, pyrophosphate

Feeding experiments with ^13^C-labeled precursors revealed anthranilic acid and acetate as molecular building blocks of aurachin D (Höfle and Kunze 2008). Cloning and gene inactivation studies were used to elucidate the biosynthesis (Fig. 1), which involves two aryl-CoA ligases, a type II polyketide synthase enzyme complex, as well as a farnesyltransferase (Sandmann et al. 2007; Pistorius et al. 2011). In native producers, such as *Stigmatella aurantiaca*, the aurachin D titer is typically below 1 mg L^−1^ (Kunze et al. 1987; Nachtigall et al. 2010). Therefore, several synthetic routes were developed to supply this bioactive agent for pharmacological testing (Li et al. 2013; Radloff et al. 2021; Dejon and Speicher 2013; Lawer et al. 2022). A biotechnological approach to generate aurachin D involves the prenylation of 2-methyl-1*H*-quinolin-4-one (MQO) with FPP in a recombinant *E. coli* strain expressing the aurachin biosynthesis enzyme AuaA (Stec et al. 2011). The conversion rate of this reaction can be substantially increased once the *E. coli* host is endowed with the non-native mevalonate pathway to enhance its metabolic flux to FPP (Kruth et al. 2022). Further possible improvements of the whole-cell biotransformation include a codon optimization of the *auaA* gene and a bicistronic design of the expression cassette. Despite these improvements, the conversion rate of MQO did not exceed 11%, corresponding to an aurachin D titer of 17 mg L^−1^ (Kruth et al. 2022).

In this study, we set out to clarify whether the toxicity of aurachin D prevented a higher product accumulation and if the biotransformation could be further improved by media optimization. Special emphasis was given to the media constituent glycerol and its impact on the whole-cell biocatalyst. Although the positive effects of glycerol on the production of recombinant proteins and terpenes in *E. coli* have been reported (e.g., in Rolf et al. 2020; Wang et al. 2022; Willrodt et al. 2014), the underlying mechanism has not yet been fully elucidated. We probed possible hypotheses, including improved substrate uptake and a growth-dependent increase in enzyme availability. Using a folding reporter, we could ultimately show that the addition of glycerol positively influenced AuaA expression, leading to an increased amount of active enzyme. Based on these findings, we also tested the replacement of the complex medium that was originally used for the biotransformation with a minimal medium. This approach not only resulted in the highest aurachin D titer ever reported but also facilitates the purification of the antibiotic from the culture broth.

## Material and methods

### Bacterial strains and cultivation conditions

All strains and plasmids used in this study are listed in Table 1. Strains were routinely grown at 37 °C in lysogeny broth (LB) medium containing tryptone (10 g L^−1^), yeast extract (5 g L^−1^), and NaCl (10 g L^−1^). The pH of the medium was adjusted to 7.0. Biotransformation studies were conducted in either terrific broth (TB) or M9 minimal medium. TB medium contained 12 g L^−1^ tryptone, 24 g L^−1^ yeast extract, 2.31 g L^−1^ KH_2_PO_4_, 12.54 g L^−1^ K_2_HPO_4,_ and 5 g L^−1^ glycerol if not indicated otherwise. The M9 medium was composed of 15 g L^−1^ glucose, 12.8 g L^−1^ Na_2_HPO_4_·7 H_2_O, 3 g L^−1^ KH_2_PO_4_, 1.0 g L^−1^ NH_4_Cl, 0.5 g L^−1^ NaCl, 0.24 g L^−1^ MgSO_4_, and 1 mL L^−1^ US* trace element solution (Panke et al. 1999). The pH of both media was adjusted to 7.2. Glucose, MgSO_4_, and the trace element solution were added as filter-sterilized solutions after autoclaving. For plasmid maintenance, the media were supplemented with ampicillin (100 µg mL^−1^), kanamycin (50 µg mL^−1^) and chloramphenicol (30 µg mL^−1^). Cultivations were carried out either on a rotary shaker (180 rpm) in baffled Erlenmeyer flasks or in a BioLector microbioreactor XT (1000 rpm) using 48-well FlowerPlates (Beckman Coulter). The FlowerPlates were sealed with gas-permeable membranes (m2p-labs) and the relative humidity of the microbioreactor was set to 80% to minimize evaporation (Rohe et al. 2012). For limonene production, the recombinant *E. coli* strain was subjected to a 50 mL two-phase cultivation in which 8 mL of diisononyl phthalate (DINP) was added at the time of induction.

**Table 1:** Strains and plasmids used in this study

Strain or plasmid	Specification	Source or reference
*E. coli* BL21(DE3)	F − *ompT hsdSB*(r_B_ − m_B_ −) *gal dcm* (DE3)	Merck KGaA
*E. coli* TOP10	F-*mcrA* Δ(*mrr-hsdRMS-mcrBC)* Φ80LacZΔM15 Δ *LacX74 recA1* araD139 Δ(*araleu)* 7697 *galU galK rpsL* (StrR) *endA1 nupG*	Invitrogen
*E. coli* SK1	*E. coli* BL21(DE3) derivative harboring pSK46_BCD9 and pJBEI-2997; used for the conversion of MQO into aurachin D	Kruth et al. (2022)
*E. coli* JK1	*E. coli* BL21(DE3) derivative harboring pJK_trGPPS_LS and pJBEI-2997; used for the production of limonene	This study
pET28a(+)	*E. coli* expression vector (pBR322 ori, f1 ori, *lacI*, *rob*, P_T7_, *lac* operator, His-Tag, T7 terminator); *kan*^R^	Novagen
pSK46_BCD9	pET28a(+) derivative for expression of *auaA* (fused to TEV-folding reporter *gfp*-His-Tag)	Kruth et al. (2022)
pJBEI-2997	*E. coli* plasmid harboring the mevalonate pathway (p15A ori, *lacI*, P_*lacUV5*_, *atoB*, HMGS, tHMGR, MK, PMK, PMD, *idi*, *ispA*); *cm*^R^	Peralta-Yahya et al. (2011)
pJBEI-6410	*E. coli* plasmid harboring the mevalonate pathway and genes for limonene production (p15A ori, P_*lacUV5*_, MTSA, T1, MBI-f, T1002, P_*trc*_, trGPPS, LS); *amp*^R^	Alonso-Gutierrez et al. (2013)
pJK_trGPPS_LS	pET28a(+) derivative for expression of trGPPS and LS; *kan*^R^	This study

*kan*^*R*^ kanamycin resistance, *cm*^*R*^ chloramphenicol resistance, *amp*^R^ ampicillin resistance

### Construction of pJK_trGPPS_LS

To generate pJK_trGPPS_LS (Figure S1), the genes trGPPS and LS were initially PCR amplified from pJBEI-6410 (Willrodt et al. 2014) using the Phusion High-Fidelity DNA polymerase (Thermo Scientific) and the primers KAG3InLimF (5′-CCGCGAAATTTGACAATTAATCATCCGGCTCGTATAATG-3′) and KAG4InLimR (5′-CCTTTCAGCGCAGAAAGGCCCACCC-3′). Afterwards, the plasmid pET28a(+) was linearized by PCR with the primers KAG1BB28aF (5′-TTTCTGCGCTGAAAGGAGGAACTATATCCGGA-3′) and KAG2BB28aR (5′-AATTGTCAAATTTCGCGGGATCGAGATCT-3′). The expression plasmid pJK_trGPPS_LS was constructed from the generated PCR products by Gibson assembly (Gibson et al. 2009). For this purpose, the 2 × GeneArt Gibson Assembly HiFi MasterMix (Invitrogen) was mixed with 100 ng of linearized plasmid and insert DNA at a ratio of 1:5 and incubated at 50 °C for 60 min. Subsequently, chemically competent *E. coli* TOP10 cells were transformed with the assembled plasmid. Sanger sequencing (Microsynth Seqlab) was used to confirm the correct sequence of pJK_trGPPS_LS.

### Determination of aurachin D and MQO toxicity

MQO was purchased from Sigma-Aldrich, while aurachin D was prepared according to a previously described protocol (Kruth et al. 2023). To determine the toxicity of both compounds, a preculture of *E. coli* BL21(DE3) was prepared in LB medium from a frozen stock culture. Following overnight cultivation in shake flasks, a fresh LB culture was inoculated with an optical density at 600 nm (OD_600_) of 0.1. This culture was grown to an OD_600_ of 1. After dilution to an OD_600_ of 0.1, 1-mL aliquots were taken and incubated for 24 h in the presence of different aurachin D concentrations ranging from 0 to 204.8 mg L^−1^. MQO toxicity was determined analogously using concentrations from 90 to 2880 mg L^−1^.

### Production of aurachin D by whole-cell biotransformation

*E. coli* SK1 was streaked on a LB agar plate and incubated overnight. A single colony was then used to inoculate a 2-mL LB culture in a 12-mL reaction tube. After incubation on a rotary shaker at 37 °C and 180 rpm for 6 h, 250 µL of this seed culture were used to inoculate a 25-mL preculture in production (i.e., TB or M9) medium. The incubation was continued overnight at 30 °C to generate inoculum for the main culture. The latter was inoculated to an OD_600_ of 0.1 and cultivated at 30 °C and 180 rpm for 48 h. If not stated otherwise, the culture volume was 50 mL and cultures were induced with 0.025 mM of isopropyl β-d-1-thiogalactopyranoside (IPTG) at an OD_600_ of 1. The substrate MQO was also added concurrently with inoculation. Microbioreactor cultures had a volume of 1 mL and were started at an OD_600_ of 0.1. Induction with 0.025 mM of IPTG was automated at a backscatter value of 275 with a gain of 5, correlating to an OD_600_ of 1.2.

### Growth and product analysis

The growth of Erlenmeyer flask cultures was quantified by measuring the OD_600_ in a spectrophotometer. The microbioreactor allowed an online monitoring of biomass by automated scattered light measurements and GFP quantification by measurement of the fluorescence (Kensy et al. 2009). The obtained data were converted into OD_600_ values and cell dry weight (CDW) concentrations (Figure S2).

Aurachin D titers were determined by HPLC analysis. For Erlenmeyer flask cultures, 1 mL of the culture was extracted three times with 400 µL ethyl acetate. The organic phases were pooled and evaporated in a Concentrator plus (Eppendorf) at 30 °C for 60 min. The residue was resuspended in 100 µL methanol and centrifuged at 13,000 × g for 10 min. The supernatant was injected into the HPLC system (Shimadzu Nexera Series LC 40) equipped with a photodiode array detector and an EC 250/4 Nucleodur C18 Isis column (250 × 4.6 mm, 5 µm, Macherey–Nagel). The mobile phase consisted of methanol (MeOH) and water with 0.1% (*v*/*v*) trifluoroacetic acid. The HPLC was operated in gradient mode at a flow rate of 0.8 mL min^−1^: 0–20 min, 40–90% MeOH; 20–30 min, 90% MeOH; 30–30.1 min, 90–40% MeOH; 30.1–35 min, 40% MeOH. The aurachin D titers were quantified by comparison with a calibration curve based on serial dilutions of a commercial aurachin D reference (AOBIOUS). Each data point represents the mean of at least two biological replicates and the error bars represent the standard deviation. For microbioreactor cultures, 500 µL of the culture was extracted and processed according to the procedure described before.

Limonene titers of the *E. coli* JK1 cultures were quantified by GC/MS analysis adapting a previously established procedure (Rolf et al. 2020). For this, the organic DINP phase was separated from the cultivation medium by centrifugation. The organic phase was diluted 1:10 with diethyl ether containing 0.2 mM *n*-dodecane (Thermo Scientific) as an internal standard and dried with MgSO_4_. Three µL of the dried sample was injected into the gas chromatograph (Shimadzu GCMS TQ-8050 NX) utilizing a Rtx-5MS column (30 m, 0.25 mm ID, 0.25 µm, Restek). The GC temperature program started at 80 °C for 5 min, before it was gradually increased to 140 °C at 7.5 °C min^−1^, and then to 300 °C at 60 °C min^−1^. The temperature was held at 300 °C for 7 min. Helium was used at a constant flow of 23.5 mL min^−1^ with a split ratio of 30. The ionization source temperature was set at 280 °C, while the interface was adjusted to 300 °C. Mass spectra were obtained in SIM mode. For limonene, the quantifier ion was *m/z* 68.0 and the qualifier ions were *m/z* 67 and *m/z* 93. For *n*-dodecane, the quantifier ion was *m/z* 57 and the qualifier ions were *m/z* 71 and *m/z* 43. The limonene titers were quantified using a calibration curve of the ratio between a commercial limonene sample (Sigma) and the internal *n*-dodecane standard.

### Quantification of glucose, glycerol, and MQO

Carbon source utilization was evaluated by analyzing culture supernatants via HPLC. For this, 300 µL aliquots of the microbioreactor cultures were centrifuged at 4 °C and 17,000 × g for 10 min to remove solids. These samples were analyzed on an Agilent 1260 Infinity HPLC system equipped with a refractive index detector (RID) and a Metab-AAC column (300 × 7.8 mm, 10 µm; ISERA). The HPLC was carried out under isocratic conditions with a mobile phase consisting of water and 5 mM sulfuric acid and a flow rate of 0.5 mL min^−1^. The column temperature was set to 40 °C.

MQO titers were determined by HPLC analysis in medium supernatant. Cells were removed with a 0.2-µm syringe filter and injected into the HPLC system (Shimadzu Nexera Series LC 40) equipped with a photodiode array detector and an EC 250/4 Nucleodur C18 Isis column (250 × 4.6 mm, 5 µm; Macherey–Nagel). The mobile phase consisted of methanol (MeOH) and water with 0.1% (*v*/*v*) trifluoroacetic acid. The HPLC was operated in gradient mode at a flow rate of 0.8 mL min^−1^: 0–5 min, 40% MeOH; 5–17 min, 40–90% MeOH; 17–22 min, 90% MeOH; 22–22.5 min, 90–40% MeOH; and 22.5–30 min, 40% MeOH. The MQO titers were quantified by comparison with a calibration curve based on serial dilutions of MQO.

### Statistical analyses

All experiments were carried out in triplicate, if not stated otherwise. Growth data and product titers were compared using one-way ANOVA (analysis of variance), followed by the post hoc Tukey test without correction for multiple comparisons. The test was conducted using OriginPro (OriginLab Cooperation). A *p*-value ≤ 0.05 was set as the threshold for statistical significance. To construct the heat maps, the mean values of two biological replicates from a previously determined set of variables were entered into the OriginPro software, and the intermediate values were interpolated using the “Color Fill Contour” tool to visualize the data.

## Results

### Product and substrate toxicity

**Fig. 2:**
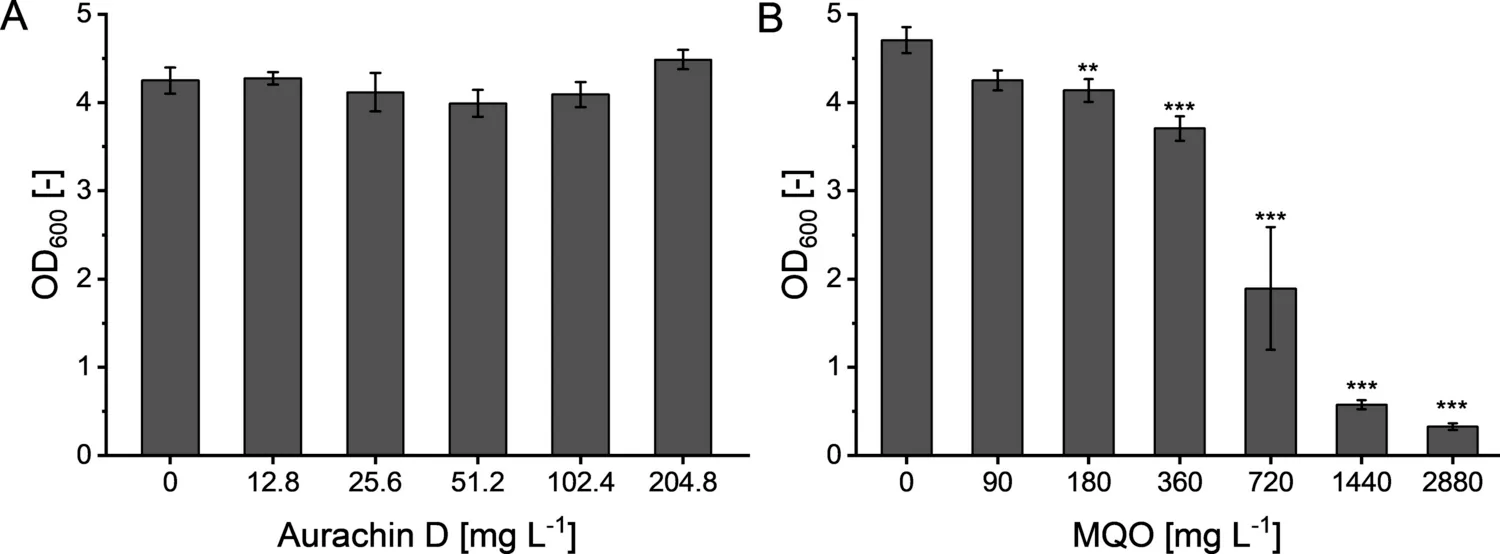
OD_600_ values of an *E. coli* BL21(DE3) culture after incubation for 24 h in the presence of different aurachin D (**A**) and MQO (**B**) concentrations. The diagram shows the mean of three biological replicates. Error bars indicate the standard deviation. ANOVA (Tukey): *p ≤ 0.05; **p ≤ 0.01; ***p ≤ 0.001 between the control (0 mg L^−1^ aurachin D or MQO) and the cultures treated with the antibiotic or the substrate, respectively. No significant differences were observed in the OD_600_ values of the cultures exposed to aurachin D

Prior to investigating the effect of medium composition on aurachin D productivity, we probed the tolerance of the recombinant host to this antibiotic and the substrate used in the biotransformation. For this purpose, we analyzed the growth of *E. coli* BL21(DE3) after incubation for 24 h in the presence of different aurachin D and MQO concentrations (Fig. 2). When exposed to aurachin D, no growth defects were observed, even at the highest tested concentration of 204.8 mg L^−1^. This result is consistent with a previous study, in which the MIC_50_ value of aurachin D against *E. coli* DH5α was reported to exceed 64 mg L^−1^ (Li et al. 2013). In contrast, the substrate MQO was found to inhibit the growth of *E. coli*, albeit only at concentrations exceeding 360 mg L^−1^. When the bacterium was incubated in the presence of 720 mg L^−1^ MQO, it reached an OD_600_ value that was only about 50% of the control. The growth further declined with increasing MQO concentrations. Noteworthy, however, no bactericidal effect was observed. Even after a 24-h incubation period in the presence of the highest MQO concentration tested, the *E. coli* cells were able to resume their growth when streaked on fresh LB agar that did not contain MQO. In sum, we concluded that the low aurachin D titers that had been previously obtained in the whole-cell biotransformation (Kruth et al. 2022) cannot be attributed to product toxicity and that it is possible to use considerably larger substrate concentrations in the biotransformation than the reported 30 mg L^−1^ MQO (Kruth et al. 2022).

### Production of aurachin D in rich media

**Fig. 3:**
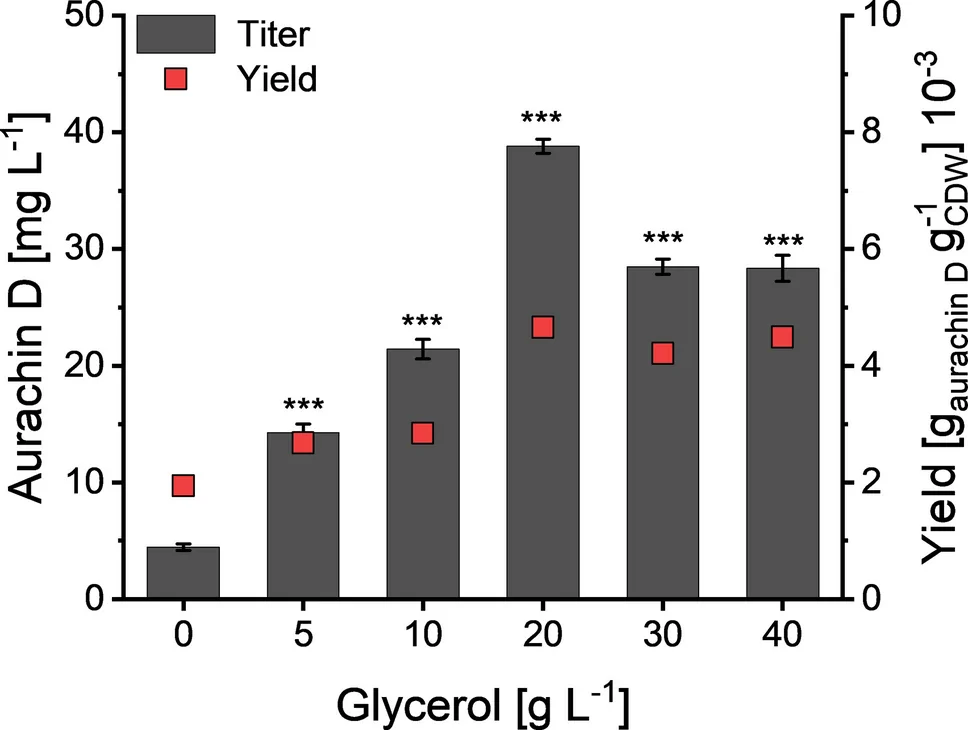
Aurachin D titers and biomass-related yields after 48-h cultivation of *E. coli* SK1 in shaken Erlenmeyer flasks containing TB medium with different glycerol concentrations. Every culture had been supplemented with 30 mg L^−1^ MQO for the bioconversion into aurachin D. The diagram shows the mean of three biological replicates. Error bars indicate the standard deviation. ANOVA (Tukey): *p ≤ 0.05; **p ≤ 0.01; ***p ≤ 0.001 between the control (0 g L^−1^ glycerol) and the cultures supplemented with glycerol

We started our analysis with the nutritionally rich TB medium, which had originally been developed to improve the yields of plasmid DNA and recombinant proteins in *E. coli* strains (Tartoff and Hobbs 1987). Both effects are due to an extended growth phase, resulting from elevated concentrations of peptone and yeast extract as well as the addition of glycerol. The latter does not only function as an extra carbon source, but it can also delay an alkalization of the culture broth, because it is metabolized by *E. coli* to acids (Kram and Finkel 2015). In light of its important role in growth and survival, we varied the glycerol concentration in TB medium and evaluated the impact on aurachin D production. This experiment indicated a positive correlation between the aurachin D titer and the amount of glycerol that had been supplied in the cultivation medium. The lowest titer of the quinolone antibiotic (4.5 ± 0.3 mg L^−1^) was obtained when glycerol was completely omitted from TB medium (Fig. 3). Using the standard glycerol concentration of 5 g L^−1^, *E. coli* reached an aurachin D titer of 14.4 ± 0.9 mg L^−1^. This value could be further improved to 38.8 ± 0.6 mg L^−1^ after increasing the glycerol content up to 20 g L^−1^. At higher glycerol concentrations, however, the aurachin D titer declined to 28.4 ± 1.1 mg L^−1^. Further experiments confirmed that the glycerol was not completely consumed when added at concentrations exceeding 20 g L^−1^ (Figure S3).

### Interrogation of glycerol effects on growth, AuaA availability, and substrate uptake

We considered various scenarios which, alone or in combination, could explain the improvement of aurachin D production in the presence of moderately elevated glycerol concentrations. One hypothesis was that more MQO had been converted into the prenylated product due to an increased availability of the whole-cell biocatalyst, resulting from an improved growth of *E. coli*. Another possibility was an enhanced expression of AuaA. Since glycerol is known to dissolve hydrophobic drugs, improving their intracellular delivery (Jiang and Williams 2022; Kawakami et al. 2006), it was further speculated whether it might also increase the cellular uptake of MQO due to a solubilizing effect.

To probe the growth hypothesis, we analyzed the specific product yield on biomass, *Y*_P/X_, calculated as the ratio of aurachin D quantity to CDW. If the availability of the whole-cell biocatalyst, i.e., the quantity of AuaA-expressing *E. coli* cells, was indeed limiting the product formation, *Y*_P/X_ should stay nearly constant at all tested glycerol concentrations. This, however, was not the case. Effectively, we observed that the *Y*_P/X_ value rose with an increasing glycerol content in the TB medium up to a concentration of 20 g L^−1^ (Fig. 3), indicating a higher conversion rate. Beyond this glycerol concentration, the aurachin D titer declined by about 25%, yet *Y*_P/X_ remained constant. We concluded that the growth-promoting effects of glycerol are not solely responsible for the elevated aurachin D titers and that the productivity gain is offset at very high glycerol concentrations.

**Fig. 4:**
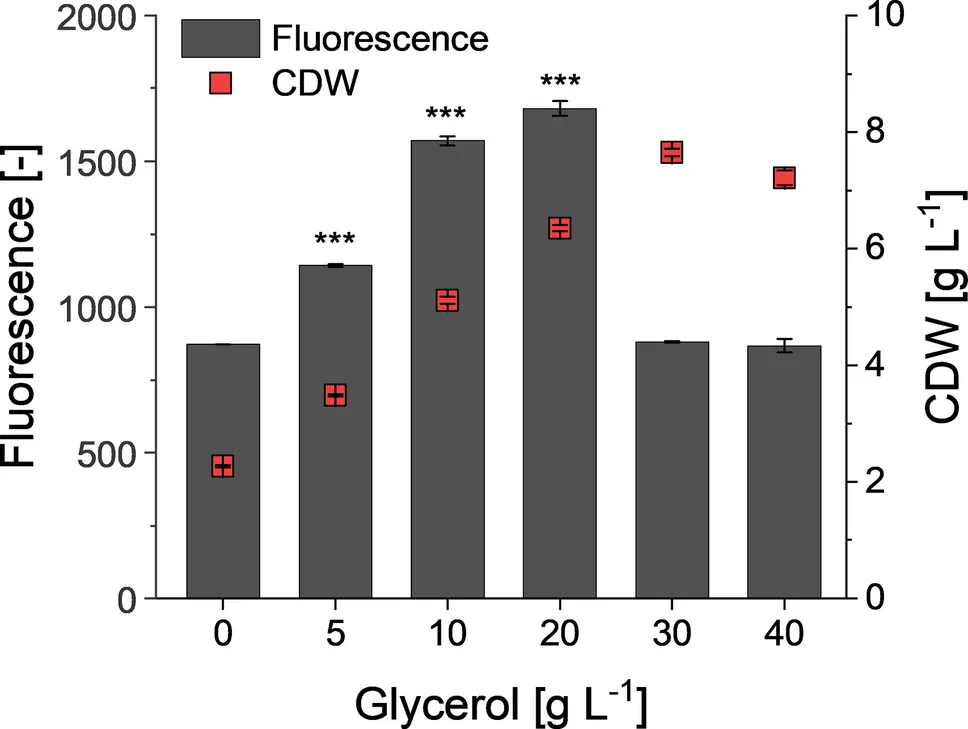
GFP fluorescence and growth of *E. coli* SK1 in the BioLector utilizing TB medium, shaking with 1000 rpm at 30 °C for 48 h. The diagram shows the mean of three biological replicates. Error bars indicate the standard deviation. ANOVA (Tukey): *p ≤ 0.05; **p ≤ 0.01; ***p ≤ 0.001 between the control (0 g L^−1^ glycerol) and the cultures supplemented with glycerol

Next, we tested whether the recombinant *E. coli* strain had become more efficient in the biocatalytic conversion of MQO due to an improved availability of active AuaA. For this, we took advantage of the fact that the *auaA* gene in the utilized expression plasmid had been fused to *gfp* (Kruth et al. 2022). This allowed an assessment of *auaA* expression by fluorescence measurements. The data obtained in this analysis was in accordance with the previously determined aurachin D titers. The fluorescence values rose up to a glycerol concentration of 20 g L^−1^, after which they decreased (Fig. 4). Since the GFP acts as a folding reporter when fused to AuaA, the fluorescence correlates with the amount of active enzyme (Drew et al. 2001). We hence reasoned that the glycerol positively influenced the AuaA expression. By increasing the glycerol concentration in the medium, the availability of active AuaA and thus the formation of aurachin D initially increased. Once a certain threshold was reached, there was no further improvement, possibly due to growth-inhibitory effects resulting from osmotic stress (Poirier et al. 1998; Mille et al. 2005).

**Fig. 5:**
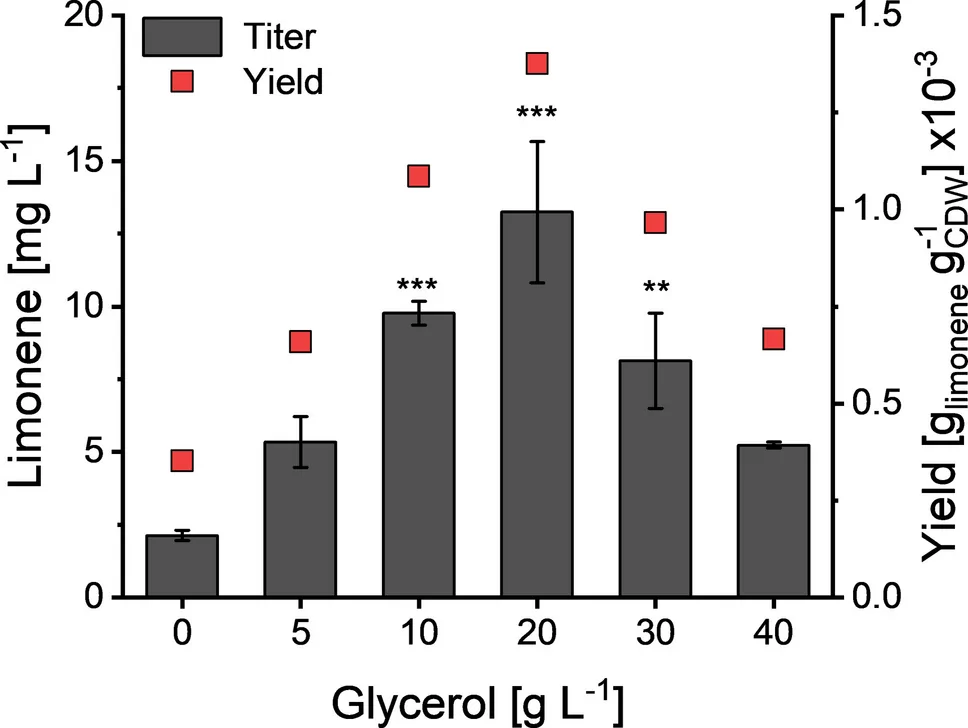
Limonene titers and biomass-related yields of *E. coli* JK1 after cultivation in TB medium with different glycerol concentrations and DINP overlay for 24 h. The diagram shows the mean of three biological replicates. Error bars indicate the standard deviation. ANOVA (Tukey): *p ≤ 0.05; **p ≤ 0.01; ***p ≤ 0.001 between the control (0 g L^−1^ glycerol) and the cultures supplemented with glycerol

To examine whether glycerol also played a role as a solubilizing agent in the cellular uptake of MQO, *E. coli* BL21(DE3) cells were suspended in PBS buffer with different glycerol concentrations and 180 mg L^−1^ of MQO. After incubation for 3 h, the MQO concentration in the supernatant was quantified. This analysis indicated that the glycerol concentration had no effect on assimilation of the fed substrate into resting cells, as consistent MQO amounts were recovered from every culture (Figure S4). Since we could not exclude the possibility of upregulated efflux pumps, we decided to analyze the impact of glycerol on the heterologous production of a terpene that does not require the cellular uptake of MQO or another substrate. For this, we constructed a limonene-producing recombinant *E. coli* strain featuring a similar genetic architecture as the aurachin D producer. The corresponding strain JK1 possessed two plasmids, including pJBEI-2997 carrying the mevalonate pathway genes (Peralta-Yahya et al. 2011) and a pET28a-derived vector for the expression of limonene synthase. After cultivation of JK1 in TB medium that had been supplemented with different amounts of glycerol, the produced limonene was quantified by GC/MS analysis. This analysis revealed that the production of limonene follows a similar pattern as aurachin D (Fig. 5). The limonene titers rose up to a glycerol concentration of 20 g L^−1^, but then dropped off. Based on the evidence gathered, the solubilization hypothesis was rejected.

### Effects of MQO on aurachin D production

**Fig. 6:**
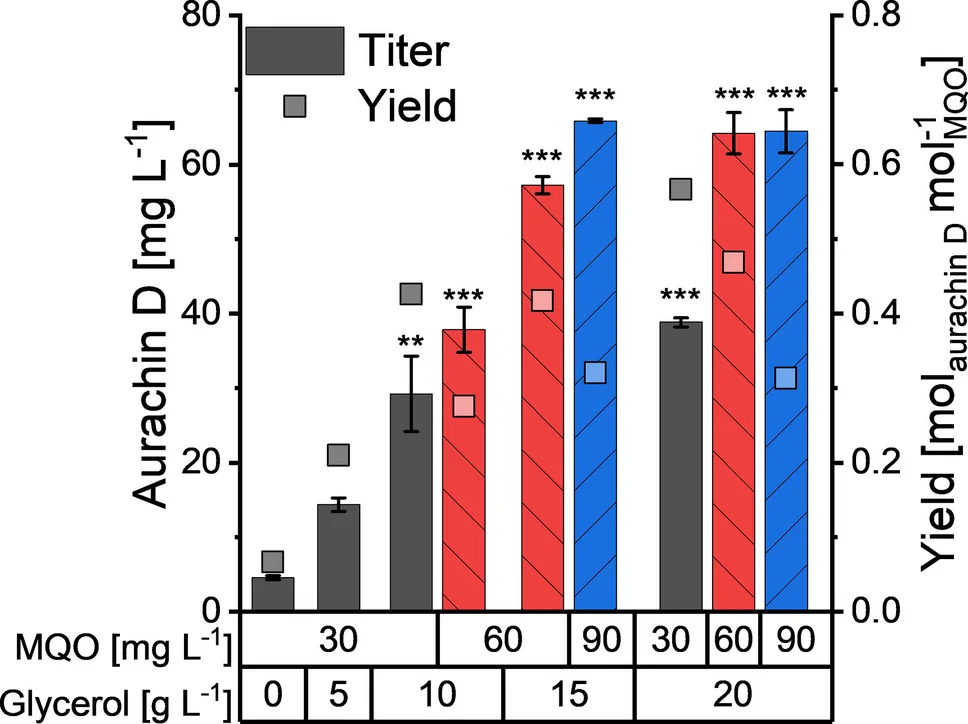
Aurachin D titers and yields after 24-h cultivation of *E. coli* SK1 in shaken Erlenmeyer flasks containing TB medium with different glycerol and MQO concentrations. The diagram shows the mean of two biological replicates. Error bars indicate the standard deviation. ANOVA (Tukey): *p ≤ 0.05; **p ≤ 0.01; ***p ≤ 0.001 between the control (0 g L^−1^ glycerol, 30 mg L^−1^ MQO) and the other cultures

After exploring the effects of glycerol on recombinant terpene production in *E. coli*, the impact of the MQO concentration on the aurachin D titer and the yield coefficient, *Y*_P/S_ (product/substrate), were evaluated in glycerol-enriched TB media. As expected, the aurachin D titer could be improved by increasing the MQO concentration, albeit at the expense of *Y*_P/S_ (Fig. 6). The observed trade-off between titer and *Y*_P/S_ is consistent with other biotransformation studies (Winand et al. 2021, 2023; Zhang et al. 2020). In the present study, a reasonable compromise was found at a glycerol concentration of 20 g L^−1^ and a MQO concentration of 60 mg L^−1^. Under these conditions, we achieved an aurachin D titer of 69.9 mg L^−1^ and a *Y*_P/S_ of 0.51 following an incubation for 48 h, corresponding to a fourfold improvement in comparison with the original process (Kruth et al. 2022).

### Production of aurachin D in minimal media

Minimal media have distinct advantages compared to rich media. Because of their defined composition, they enable more reproducible fermentation performance and give consistent results at different scales (Müller et al. 2018). Minimal media also avoid the risk of inflating yield calculations by neglecting unquantified carbon and nitrogen sources in complex media formulations (Yook and Alper 2025). Moreover, they simplify the downstream processing of products in culture supernatants. Due to these beneficial traits, we tested the biocatalytic production of aurachin D in the well-known M9 mineral medium, which contains glucose as sole carbon source (Panke et al. 1999). Initial experiments led to lower product titers than those obtained in the rich TB medium. Notably, the *E. coli* host SK1 also produced less biomass and showed a delayed growth in M9 medium. The CDW was 1.43 g L^−1^ compared to the 7.56 ± 0.12 g L^−1^ in TB medium after 24 h of incubation. On the other hand, the specific aurachin D yield on biomass was increased in M9 medium. This indicated that more carbon had been channeled towards product formation instead of being used for biomass production.

**Fig. 7:**
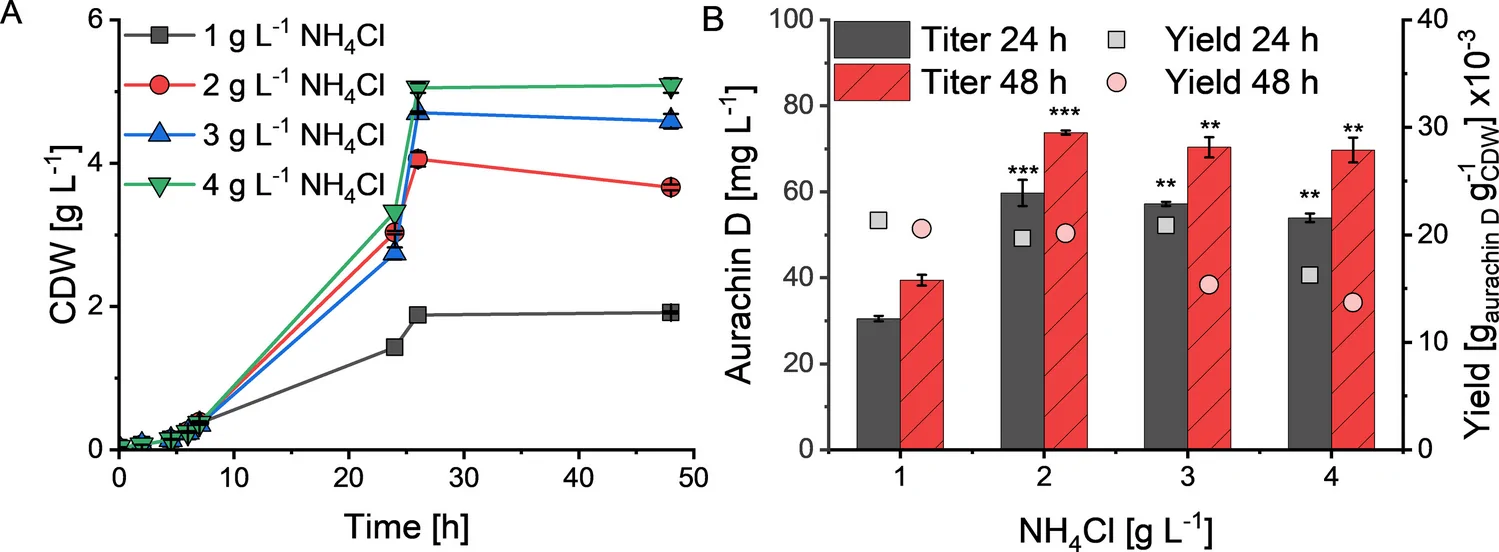
CDW values (**A**), aurachin D titers and yields (product/biomass) (**B**) after batch cultivation of *E. coli* SK1 for 24 h and 48 h in M9 medium containing 15 g L^−1^ glucose and different NH_4_Cl concentrations. All cultivations were carried out in baffled Erlenmeyer flasks at 30 °C and 180 rpm. The diagram shows the mean of two biological replicates. Error bars indicate the standard deviation. ANOVA (Tukey): *p ≤ 0.05; **p ≤ 0.01; ***p ≤ 0.001 between the control (1 g L^−1^ NH_4_Cl) and the other cultures

Literature precedence suggested that the reduced growth is mainly due to nitrogen limitation (Nerke et al. 2024). Hence, the nitrogen content in the minimal medium was varied and the effects on the growth of the production host, the aurachin D titer, and the product yield were evaluated. This experiment showed that the growth of *E. coli* SK1 favorably responds to an increase of the nitrogen concentration in M9 medium. While the maximum CDW was reached at the highest NH_4_Cl concentration tested (4 g L^−1^), the highest aurachin D titer and yield were obtained with an NH_4_Cl concentration of 2 g L^−1^ (Fig. 7). This amount of NH_4_Cl was hence used in all subsequent cultivation studies. It is further noteworthy, that in M9 medium higher aurachin D titers were consistently obtained after extending the cultivation time to 48 h. Just like with the TB cultures, it was possible to improve the product titer by increasing the MQO concentration in M9 medium (Figure S5). Here, the highest titer was 104.2 ± 1.3 mg L^−1^ following an incubation for 24 h and a MQO starting concentration of 180 mg L^−1^.

**Fig. 8:**
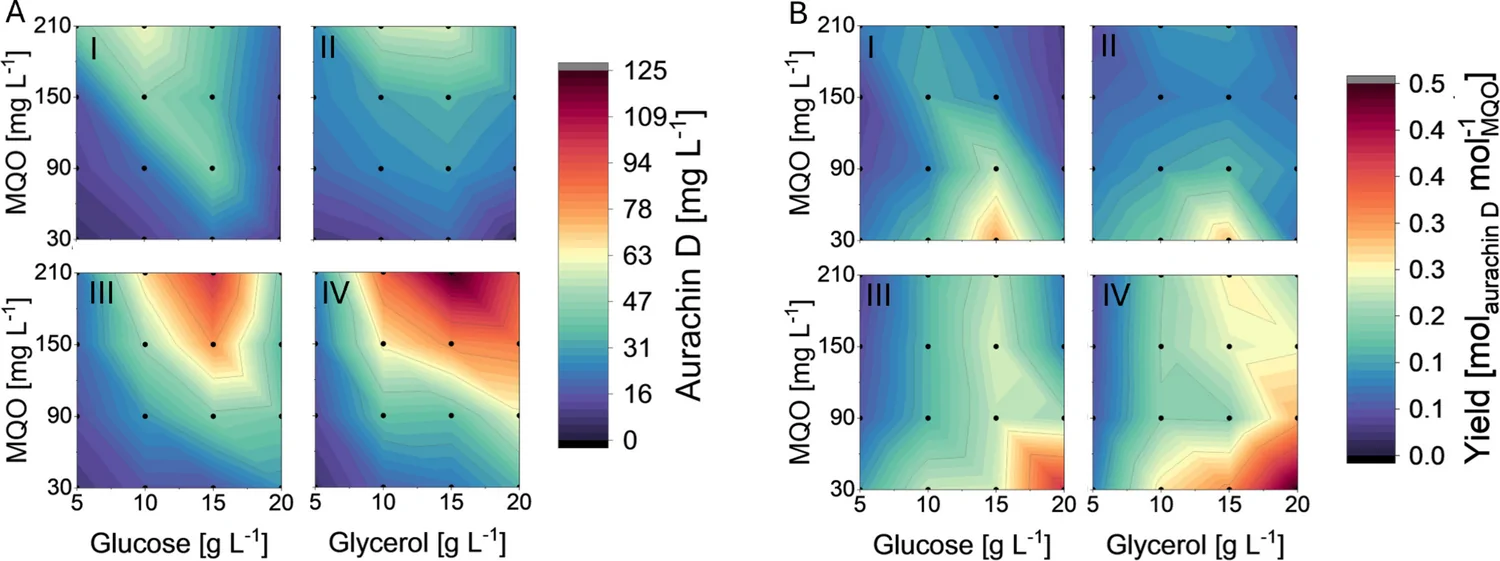
Heat maps of aurachin D titers (**A**) and yields (**B**) after a 48-h batch cultivation of *E. coli* SK1 in a microbioreactor system at 30 °C and 1200 rpm. Glucose and glycerol served as supplements in TB medium (I and II, respectively), whereas they constituted the sole carbon sources in M9 medium (III and IV, respectively). The measurement points are marked with black dots and represent the mean of two biological replicates

Given the positive effects of glycerol in TB medium, we eventually probed its use as a surrogate for glucose in M9 medium with 0.2% NH_4_Cl. For an unbiased comparison of the different carbon sources, we also tested the supplementation of TB medium with glucose. The corresponding biotransformation studies were carried out in a microbioreactor system to enhance their reproducibility (especially with regard to mixing and aeration) and provide a data basis for process development. The results of these experiments are depicted in Fig. 8. Additional data on the fluorescence and growth of *E. coli* SK1 are found in Figures S6 and Figure S7. According to these studies, a moderate increase in glycerol concentration generally has a positive effect on aurachin D titers and MQO conversion rates. This effect is not limited to rich TB medium but also occurs in M9 medium. A similar, albeit less pronounced increase was observed with glucose supplementation. Overall, the defined M9 medium proved to be superior to the TB medium. The highest aurachin D titer was measured in M9 medium with a glycerol content of 15 g L^−1^ (124 mg L^−1^ *vs.* 61.5 mg L^−1^ in TB medium under the same conditions). The heat map suggests that even higher aurachin D titers can be achieved by further increasing the MQO concentration. However, given the low substrate-dependent yield, this approach was not pursued further.

## Discussion

Substrate and product toxicity are negligible in the production of aurachin D with recombinant *E. coli* cells, whereas the media composition was demonstrated to have a considerable effect on the productivity in a batch process. Especially the use of glycerol as supplementary or sole carbon source promoted the biosynthesis of aurachin D from MQO. Experimental evidence suggests that the beneficial effect of glycerol is due more to increased availability of the biosynthesis enzyme AuaA than to improved growth. Once a certain threshold value for glycerol concentration is exceeded, both the amount of active enzyme and the productivity decline, which can be explained with osmotic stress.

Glycerol is well-known to stabilize enzymes in vitro by acting as a cosolvent, reducing water activity and preferentially interacting with protein surfaces to inhibit unfolding, preventing aggregation, and maintaining native conformation (Vagenende et al. 2009). On the other hand, information on the effects of glycerol on enzymes under in vivo conditions is sparse. Previous studies on human phenylalanine hydroxylase and glutathione-*S*-transferase showed that their expression in *E. coli* yielded more active enzyme when the host was grown in the presence of glycerol (Leandro et al. 2001; Hansen and Eriksen 2007). In both cases it was speculated that the glycerol must have corrected protein folding abnormalities. To our knowledge, experimental support for this assumption has been missing, but now comes from our analysis using the GFP folding reporter.

The results of the present study further showed that productivity improvements are possible by switching from a rich to a minimal medium. The latter also facilitates the isolation of aurachin D from the culture broth. Another possible parameter for adjustment is the concentration of the substrate used in the biotransformation. Higher MQO concentrations boost the aurachin D titer but, at the same time, reduce the specific yield. Future work should thus focus on the bioprocess operation mode and evaluate the production of aurachin D under fed-batch conditions and/or in a two-phase system in order to improve the conversion rate. Overall, this study underscores the importance of considering both the biocatalyst and the cultivation conditions when optimizing whole-cell processes (Willrodt et al. 2014).

## Supplementary Information

Below is the link to the electronic supplementary material.

ESM 1 (PDF 460 KB)

## Data Availability

The plasmids, strains, and datasets generated are available from the corresponding author upon request. Additional information is provided in the Supplementary Material.
