# Supplementary material for: Glycerol-mediated improvement of heterologous aurachin D production in E. coli
Source: Appl Microbiol Biotechnol. 2026 May 30;110(1):164. doi: 10.1007/s00253-026-13890-2 (PMC13222167; doi:10.1007/s00253-026-13890-2)
Supplement: Supplementary file 1 — (PDF 460 KB) [file 253_2026_13890_MOESM1_ESM.pdf]

Supplementary Information

Glycerol-mediated improvement of heterologous aurachin D production in *E. coli*

Jonas Korb, Büsra Demir, Kai Graw, Markus Nett

TU Dortmund University, Department of Biochemical and Chemical Engineering, Dortmund, Germany

e-mail: markus.nett@tu-dortmund.de

Table of contents

Figure S1. Plasmid map of pJK_trGGPS_LS……………………………………………………………………………………….SI-2

Figure S2. Conversion of backscatter values from scattered light measurements……………..……………..…….SI-3

Figure S3. Recovery of glycerol after cultivation of *E. coli* SK1 in TB medium for 24 h………………………….SI-3

Figure S4. Recovery of MQO from *E. coli* BL21(DE3) maintained in PBS buffer……….…………………….……….SI-4

Figure S5. Aurachin D titers in M9 medium containing different MQO concentrations……………………….SI-4

Figure S6. Fluorescence and backscatter values of *E. coli* SK1 in TB medium…………….………………………….SI-5

Figure S7. Growth curves of *E. coli* SK1 measured in a BioLector microbioreactor XT system………………SI-6

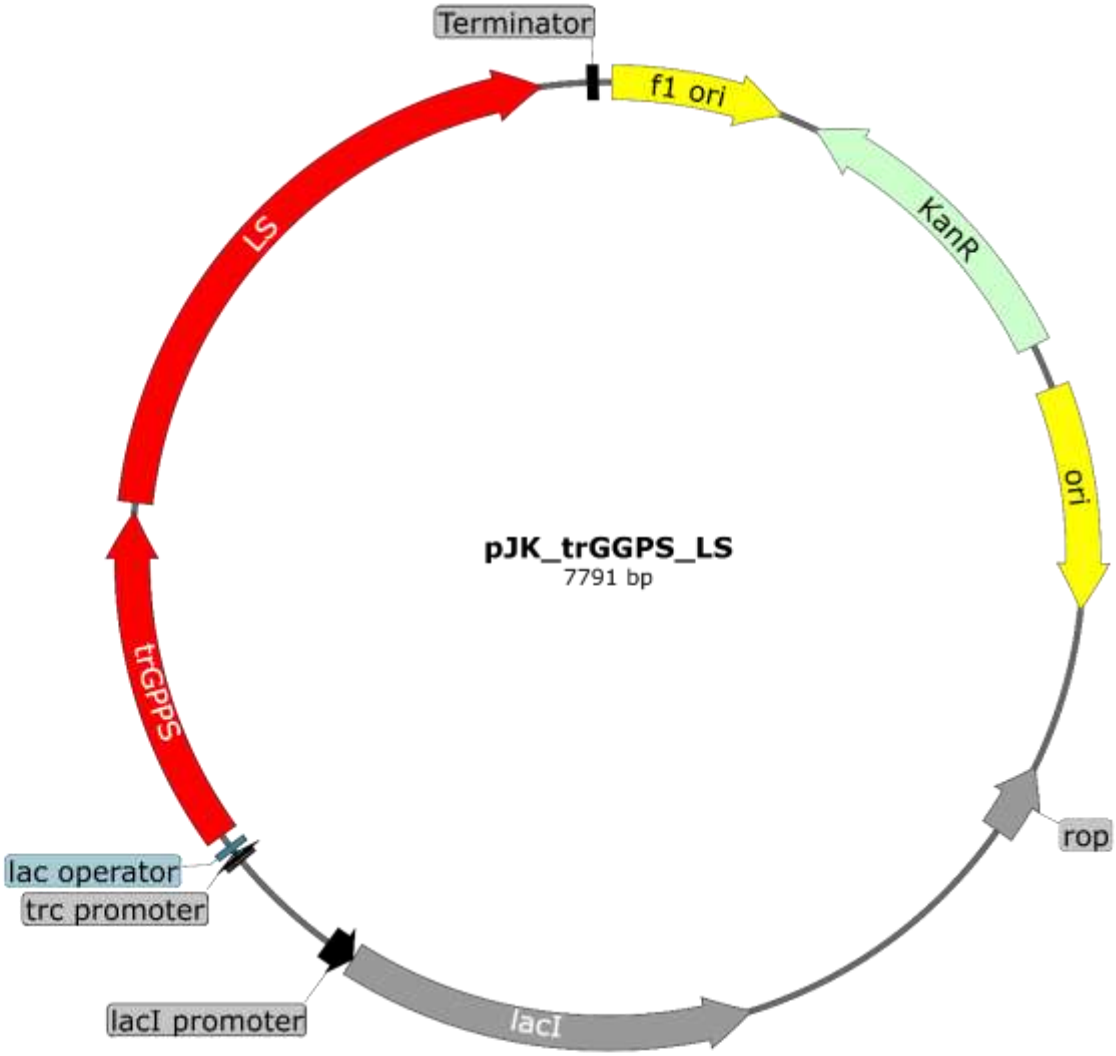

Figure S1. Plasmid map of pJK_trGGPS_LS. The genes depicted in red were inserted into the pET28a(+) backbone by Gibson assembly.

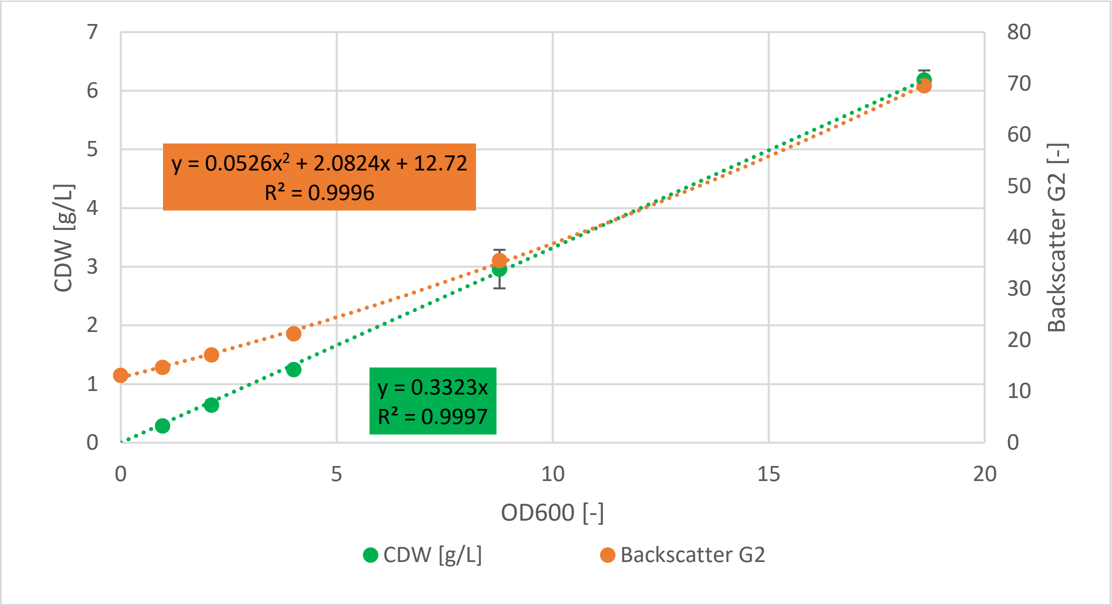

Figure S2. Conversion of backscatter values from scattered light measurements into OD_{600} values and CDW concentrations. The backscatter values and CDW concentrations were measured using an *E. coli* BL21(DE3) culture that was diluted to different OD_{600} values. The diagram shows the mean of three biological replicates. Error bars indicate the standard deviation.

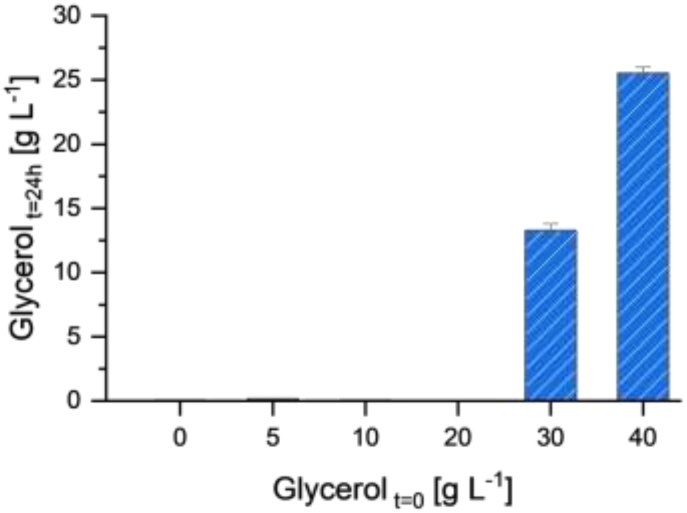

Figure S3. Recovery of glycerol after cultivation of *E. coli* SK1 in TB medium for 24 h. Every culture had been supplemented with 30 mg L^{-1} MQO and different concentrations of glycerol. The diagram shows the mean of three biological replicates. Error bars indicate the standard deviation.

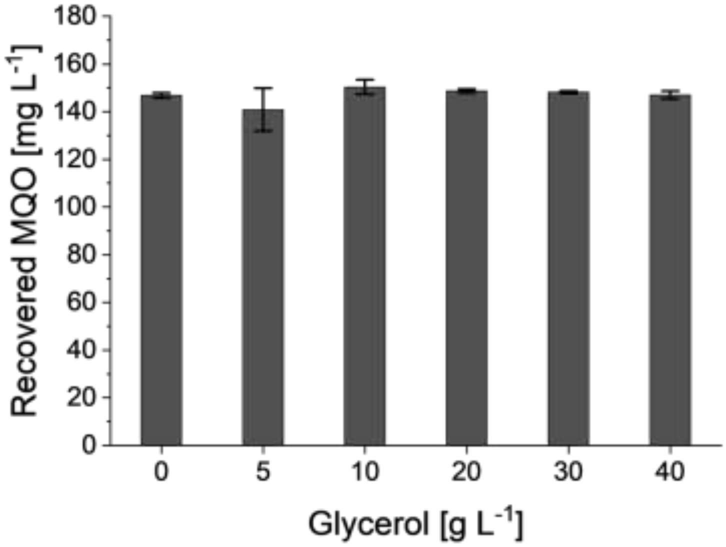

Figure S4. Recovery of MQO from *E. coli* BL21(DE3) maintained in PBS buffer following an incubation for 3 h at 30 °C. Prior to the incubation, the PBS buffer was supplemented with 180 mg L^{-1} of MQO. The diagram shows the mean of three biological replicates. Error bars indicate the standard deviation.

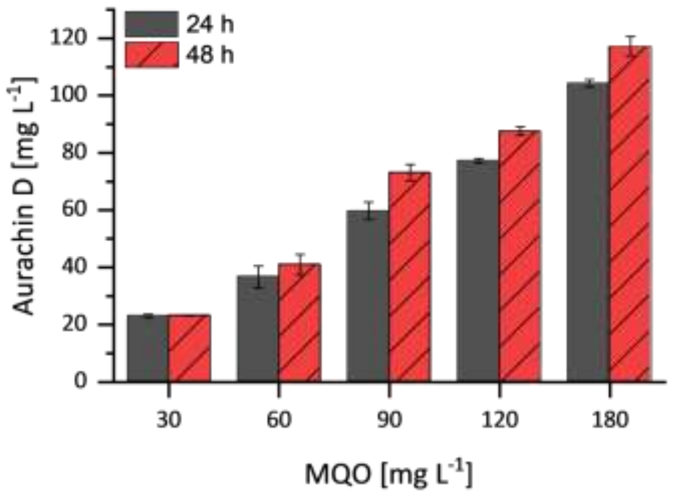

Figure S5. Aurachin D titers after cultivation of *E. coli* SK1 in M9 medium containing 15 g L^{-1} glucose and different MQO concentrations. All cultivations were carried out in baffled Erlenmeyer flasks at 30 °C and 180 rpm. The diagram shows the mean of two biological replicates. Error bars indicate the standard deviation.

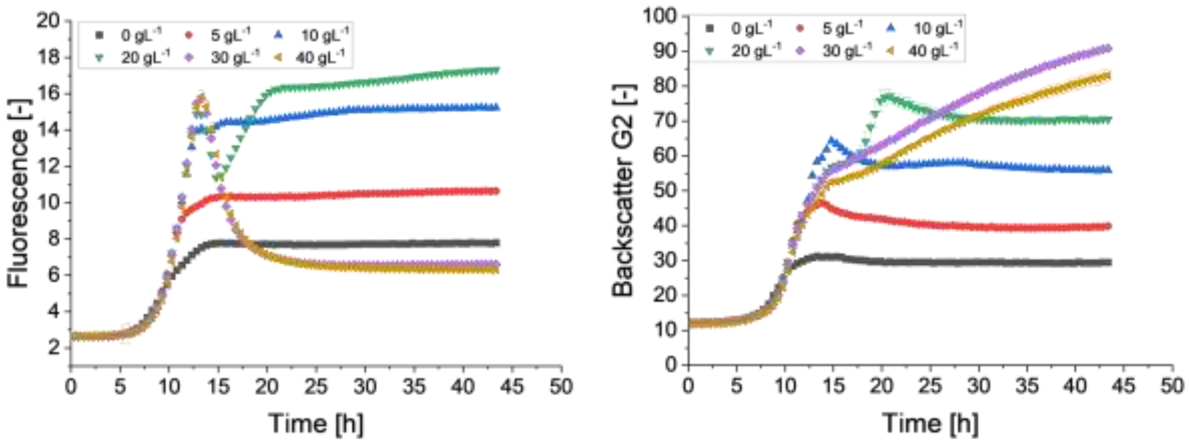

Figure S6. Fluorescence and backscatter values of *E. coli* SK1 in TB medium with different glycerol concentrations. The cultivation was carried out in a microbioreactor system at 30°C and 1,000 rpm. For induction, 0.25 mM IPTG was added at an OD_{600} of 1. The diagram shows the mean of three biological replicates.

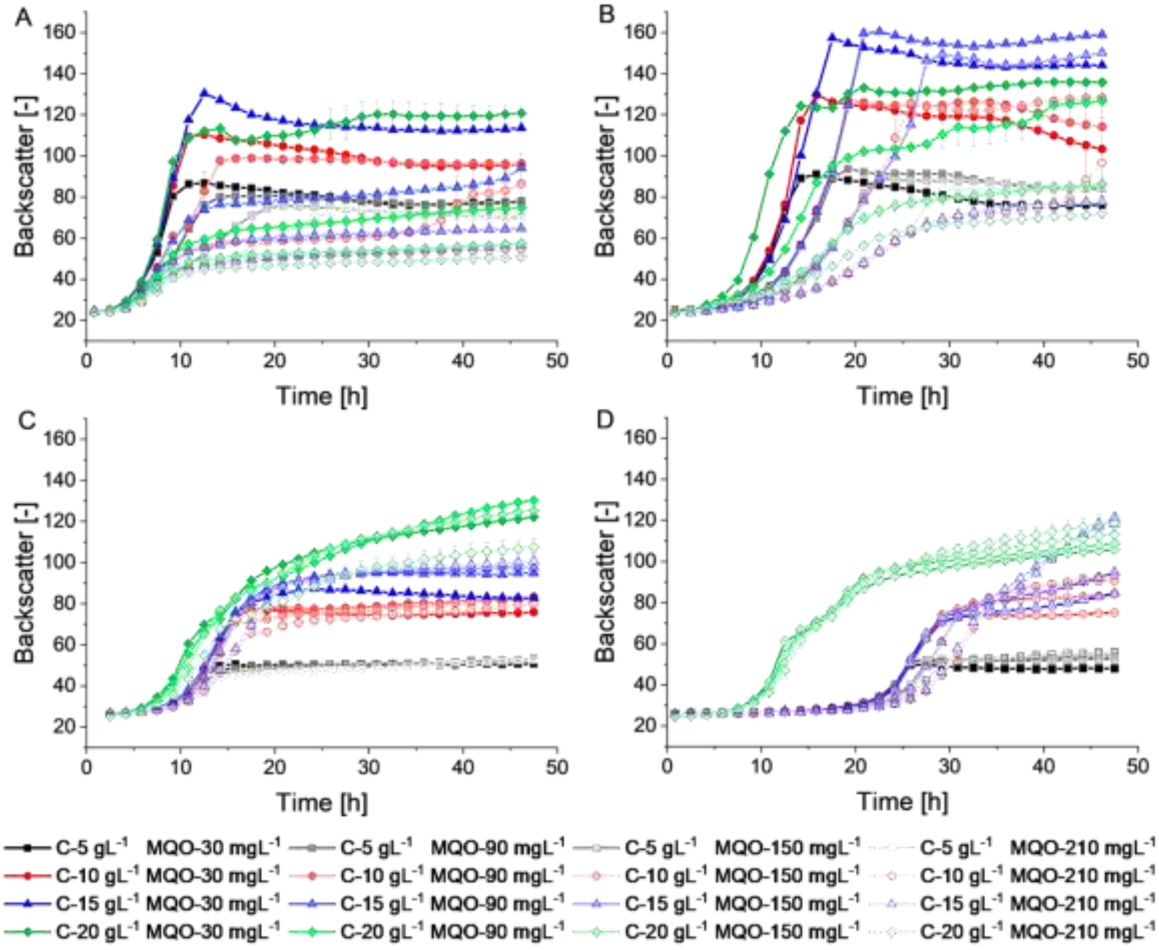

Figure S7. Growth curves of *E. coli* SK1 were measured in a microbioreactor system at 30°C and 1,200 rpm at gain 3. The cultivations were conducted in TB medium containing either glucose (A) or glycerol (B), as well as M9 medium containing either glucose (C) or glycerol (D). The diagram shows the mean of three biological replicates.
